# Extracellular vesicles as mediators of exercise effects in cancer

**DOI:** 10.1186/s10020-026-01577-3

**Published:** 2026-08-01

**Authors:** Inês Aires, Daniel Moreira-Gonçalves, Vito G. D’Agostino, Rita Ferreira, Rui Vitorino

**Affiliations:** 1 Department of Chemistry, LAQV-REQUIMTE, University of Aveiro, Aveiro, 3810-193 Portugal; 2 Department of Medical Sciences, Institute of Biomedicine iBiMED, University of Aveiro, Aveiro, 3810-193 Portugal; 3 Research Centre in Physical Activity, Health and Leisure (CIAFEL) and Laboratory for Integrative and Translational Research in Population Health (ITR), Faculty of Sports, University of Porto (FADEUP), Porto, 4200-450 Portugal; 4 Department of Cellular, Computational and Integrative Biology (CIBIO), University of Trento, Via Sommarive 9, Trento, 38123 Italy

**Keywords:** Aerobic exercise, Resistance exercise, Extracellular vesicles, Exerkines, Cancer

## Abstract

Exercise is a well-established non-pharmacological intervention in oncology, consistently associated with reduced cancer risk and improved disease outcomes. Yet, the molecular mechanisms through which physical activity exerts its anticancer effects remain incompletely understood. Extracellular vesicles (EVs) released in response to exercise have emerged as promising candidate mediators of these effects, acting as intercellular communication carriers that transport bioactive molecules modulating the tumor microenvironment and capable of interacting with distant tissues. The composition of EV cargo is highly sensitive to exercise modality, intensity, and physiological status. Aerobic and resistance exercise each elicit different EV molecular signatures that converge on key processes, including angiogenesis, immune surveillance, metabolic reprogramming, and inflammatory signaling. Preclinical data indicates that EVs obtained from exercised animals reduce tumor cell viability, impair proliferation and migration, induce apoptosis, and slow tumor progression in sedentary animals, positioning EVs as key mediators of exercise-induced tumor suppression. However, the contribution of different cellular origins, exercise protocol specificity, as well as biological sex to EV composition and anticancer function remain largely unresolved, limiting mechanistic interpretation and clinical translation. This review synthetizes the current evidence on the role of EVs released in response to exercise in cancer development and progression, identifies the key biological and methodological gaps impeding progress, and evaluates the translational challenges that must be overcome to harness their therapeutic potential in oncology.

## Introduction

Despite the well-established health benefits of exercise, around one third of adults worldwide remain physically inactive, and this proportion is projected to rise up to 35% by 2030 (Hallal et al. 2012, Haileamlak 2019, Hawley and Holloszy 2009). Several factors appear to drive this trend, including car-centric environments, sedentary work and screen-based leisure, combined with low motivation or self-confidence to engage in exercise (Evans et al. 2022). Physical activity not only helps prevent noncommunicable diseases such as cancer, but also has therapeutic value, reducing treatment side effects, improving quality of life, and potentially lowering recurrence and mortality in people living with cancer (The Lancet Oncology 2018, Hojman et al. 2018). However, questions about how to optimize the benefits of exercise in cancer care remain to be clarified, including the most effective duration, intensity, and type of exercise, and identifying the best approach for each individual is often challenging. Many patients, particularly those with advanced diseases and multiple comorbidities, struggle to participate in exercise programs, which limit their ability to fully realize the health benefits that physical activity could specifically provide. Even among those who do exercise during cancer treatment, cancer-related fatigue, pain and reduced mobility are common barriers to regular participation. To address these challenges, individualized exercise prescriptions, close supervision, and gradual progression of intensity are crucial to promote adherence while maximizing safety and therapeutic effectiveness (Schumann et al. 2020, Thomas 2022). Optimizing these prescriptions requires a precise understanding of how exercise stimuli translate into physiological and molecular adaptations in the cancer context. Regular exercise has been shown to reduce systemic inflammation, modulate insulin and IGF-1 signaling, enhance NK cell mobilization and cytotoxic T lymphocyte infiltration into tumors (Wennerberg et al. 2020, Gomes-Santos et al. 2021), and promote the release of myokines with direct anti-proliferative and pro-apoptotic effects on cancer cells (Kleckner et al. 2019, Huang et al. 2022). Furthermore, exercise may also promote the remodeling of tumor vascularization (Faustino-Rocha et al. 2016) and alter tumor metabolism by modifying lactate dynamics, improving perfusion, and enhancing oxygen delivery (Vulczak et al. 2020). These effects are, however, dependent on exercise parameters, including modality, intensity, volume and timing relative to diagnosis and treatment, as well as host-related factors such as cancer type and stage, treatment regiment, age, metabolic status and baseline fitness level (Kang et al. 2026, Campbell et al. 2019). The mechanisms through which specific exercise stimuli are sensed, transduced, and communicated to the tumor microenvironment remain incompletely understood, a gap that underscores the need to dissect how exercise signals are encoded at their tissue of origin, transmitted through the circulation and decoded by tumor and immune cells to produce the observed biological effects.

Central to this exercise-induced inter-organ and host-tumor communication are exerkines, bioactive molecules (such as proteins like myokines, nucleic acids, lipids and metabolites) released into the circulation by various tissues, primarily skeletal muscle, but also adipose tissue, liver, heart and others, in response to acute or chronic exercise, with paracrine and endocrine signaling capacity (Chow et al. 2022). Whether these molecules act as free circulating mediators or are selectively packaged within extracellular vesicles (EVs) remains largely unresolved.

EVs have emerged as compelling candidates for mediating exercise-induced inter-organ communication because they circulate in biofluids, carry diverse bioactive cargo protecting them from enzymatic degradation, capable of crossing biological barriers, and able to reprogram recipient cells within the tumor microenvironment (Darkwah et al. 2021, Maacha et al. 2019, Puurand et al. 2025). By modulating EV release, molecular cargo, and uptake, exercise influences cancer-relevant processes like inflammation, angiogenesis, and immune responses (Mlynska et al. 2025). However, EV release, concentration and cargo composition vary with exercise modality (aerobic vs. resistance), intensity and duration, underscoring their dynamic role in exercise-driven adaptations. In this review, we focus on exercise-induced EVs in circulation as key mediators of tissue crosstalk, exploring how their molecular cargo may drive the anticancer effects of exercise and responses to therapy.

## Exercise-induced changes in extracellular vesicle release and molecular content

EVs are lipid bilayer‑enclosed particles released by various cell types into the biofluids, serving as mediators in intercellular communication as they transport molecules (nucleic acids, proteins, lipids and other metabolites) between donor and recipient cells, both locally and systemically (Whitham et al. 2018). EVs released into blood circulation can originate from various tissues and are often identified according to their molecular cargo and surface markers, being highly heterogeneous with both small and large diameters (Welsh et al. 2024). According to “Minimal Information for Studies of Extracellular Vesicles” (MISEV2023) guidelines, published by the International Society for Extracellular Vesicles (ISEV), EVs are operationally classified into small EVs (< 200 nm) and large EVs (> 200 nm) based on size-based separation methods such as differential ultracentrifugation (Welsh et al. 2024). However, researchers should be aware of their limitations, as no strict consensus exists on size cut-offs and many separation methods yield EV populations with overlapping size profiles. Importantly, size alone does not inform on EV biogenesis or cellular origin, and operational terms should be clearly defined with the context of the separation method used. ISEV discourages the use of the term “exosome” unless subcellular origin can be demonstrated (Welsh et al. 2024). When such confirmation is available, EVs may be described as exosomes (50–200 nm) formed within the endolysosomal system or by direct budding from the plasma membrane. Microvesicles (0.1–1 μm) originate through outward budding of the plasma membrane, often involving cytoskeletal rearrangements. Whereas, apoptotic bodies (100–5 μm) are released from dying cells during apoptosis and contain cytoplasmic organelles or nuclear fragments, are subsequently recognized and cleared by macrophages (Rädler et al. 2023). The distinct biogenesis pathways of each EV subpopulation significantly influence their molecular cargo enrichment, and consequently their biological function, enabling tissue-specific signaling (Colombo et al. 2014, Wakker et al. 2024). An accurate determination of EV size and markers is essential for their proper characterization and to distinguish EVs from contaminants (e.g. lipoproteins, protein aggregates), as recommended by the ISEV (Théry et al. 2018). The reporting of both particle size/concentration (via nanoparticle tracking analysis or dynamic light scattering) and canonical tetraspanin markers such as CD9, CD63, and CD81, alongside absence of non-EV markers, ensures identification and reproducibility. Because current isolation techniques (e.g. ultracentrifugation or size-exclusion chromatography) yield heterogeneous populations, the terms small and large EVs are recommended, but should be used with caution because they can be influenced by separation methods (Welsh et al. 2024). Current EV isolation techniques include ultracentrifugation, size exclusion chromatography, polymer precipitation, electrostatic interaction, and affinity enrichment, with the first three being the most commonly used (Comprehensive, 2025). The choice of the isolation technique directly influences the purity and yield of EVs introducing important variability across studies. Ultracentrifugation is the most widely used but may isolate contaminants and physically disrupt vesicle membranes. Size exclusion chromatography offers a higher purity and better EV preservation, though requires a higher investment per sample compared to other techniques. Precipitation-based methods (e.g., ExoQuick) offer simplicity and high particle yield but are susceptible to co-isolation of free RNAs and non-vesicular proteins (Zhao et al. 2021, Monguió-Tortajada et al. 2019). The lack of standardization for techniques to isolate EV, limits direct comparison across studies and underscores the importance of reporting, as recommended by ISEV (Saint-Pol and Culot 2025).

### Cellular origin and target of exercise-induced EVs

A consistent fraction of circulating EVs are platelet-derived (CD41⁺, CD42⁺ and CD62⁺) accounting for 70–90%, depending on the platelet stimuli (Eustes and Dayal 2022). The remaining EV population are derived from peripheral blood mononuclear cells (PBMCs; CD4⁺/CD8⁺ lymphocytes, CD14⁺ monocytes) and endothelial cells (CD105⁺, CD146⁺), while skeletal muscle-derived EVs (SGCA⁺) represent only approximately 5% of total circulating EVs, despite skeletal muscle comprising ~ 40% of body mass and being recognized as an endocrine organ (Guescini et al. 2015, Aatonen et al. 2014, Nederveen et al. 2021). Plasma is preferred over serum for EV isolation, as clotting activates platelets and artificially inflates their EV contribution (Taha 2023), potentially masking subtler exercise-induced signals from other tissues. Exercise does not substantially alter this hierarchy with platelets and immune cells remaining the major contributors to the circulating EV pool post-exercise, with muscle EVs showing minimal quantitative increase (Kargl et al. 2024). Although immune cells constitute the main origin of exercise-induced EVs, the mechanisms linking exercise-induced immune activation to EV release, remain poorly understood. Moreover, the timing of post-exercise sample collection critically influences both EV concentration and molecular cargo, likely reflecting dynamic, cell type-specific contributions to the circulating EV pool. Acute exercise triggers rapid platelet and endothelial EV release within minutes to hours (peaking ~ 1–2 h post-exercise), while muscle- and immune cell-derived EVs may rise later (4–24 h), carrying distinct cargo signatures tied to metabolic stress, myokine production, or immune activation (Nederveen et al. 2021, Maggio et al. 2023, Frühbeis et al. 2015). This temporal heterogeneity underscores the need for standardized sampling protocols to dissect exercise-induced EVs shifts accurately in oncology studies.

The contribution of exercise-induced skeletal muscle to circulating EVs is dependent on the exercise protocol. Acute resistance exercise has been associated with increased circulating EVs carrying muscle-associated markers, compared to other exercise protocols. Indeed, a single bout of resistance exercise (5 sets of 10 maximal squats on a Flywheel) showed to stimulate the secretion of muscle-derived EVs, encapsulated with miR-206 (approximately 2-fold, compared to before exercise) (Annibalini et al. 2019), whereas a single-bout of endurance training (cycling for 60 min at 55% VO_2_max) failed to alter the levels of proteins involved in EV formation such as ALIX1, TSG101, CD9 and CD81, which were measured in muscle samples, suggesting no acute increase in intramuscular EV biogenesis (Warnier et al. 2022). This protocol dependency may reflect differences in recruited muscle fiber type, with oxidative fibers releasing higher amounts of EV into circulation compared to glycolytic fibers (Nie et al. 2019). Importantly, even with these modulations of skeletal muscle secretory activity, platelets and immune cells remain the dominant sources of the post-exercise circulating EV pool (Brahmer et al. 2019).

The ability of EVs to exert organ- and cell-specific effects depends on their recognition and interaction with recipient cells. The target specificity is mediated by adhesion molecules present on EV surface that act as a molecular ‘postal code’, directing them to compatible recipient cells via receptor-ligand interactions (Raposo and Stoorvogel 2013, Denham and Spencer 2020). Key players include integrins, selectins, ICAM-1/VCAM-1, and tetraspanins (CD9+/CD63+/CD81+), which reflect the EV cell of origin and target compatible cells (Ginini et al. 2022). EV uptake by recipient cells occurs mainly via endocytic pathways, including clathrin-dependent endocytosis, caveolin-mediated uptake, macropinocytosis, and lipid raft-mediated internalization (Montecalvo et al. 2012, Mulcahy et al. 2014). Additionally, direct membrane fusion may also occur, particularly when EVs interact with plasma membrane receptors or cells with phagocytic capacity (such as macrophages). Importantly, EV uptake is an energy-dependent process, which requires a functioning cytoskeleton (Ginini et al. 2022, Mulcahy et al. 2014). After being internalized, and depending on their route of internalization, membrane fusion or endocytosis, EV content will be released either directly into the cytoplasm, in case of membrane fusion, or into endosomal compartments, in case of endocytosis. Additionally, EVs transporting miRs may have nuclear signals that enable nuclear translocation and epigenetic effects on target cells. These mechanisms can vary depending on the target cell, EV molecular cargo and EV surface markers (Jia et al. 2025).

Exercise can enhance this targeting specificity by modulating EV surface adhesion proteins. Following a single-bout endurance protocol (cycling for 60 min: 30 min at 55% VO_2_max, 20 min at 70% and 10 min at 80%), circulating EVs showed a significant increase in adhesion proteins such as ICAM-1, VCAM-1 and ITGB5, which were subsequently detected within the liver, supporting organ-specific homing. ITGB5 on exercise-induced EV surface acts as a molecular ‘liver postal code’, binding hepatic receptors to direct metabolic signaling to this organ (Whitham et al. 2018). Following internalization in target cells, EV molecular cargo mediate a range of exercise-induced effects including tissue repair, immune regulation, and metabolic adaptation (Puurand et al. 2025, Nederveen et al. 2021).

### Exercise modality-dependent profile of circulating EVs

Different exercise modalities regulate both the quantity and molecular cargo of circulating EVs, creating distinct ‘EV fingerprints’ that are likely to contribute to modality-specific adaptations.

In a rat treadmill study (40 min/day for one week at low-intensity: 14–16 m/min, moderate-intensity: 20–22 m/min and high-intensity: 24–26 m/min), high-intensity running was associated with a shift toward smaller EVs, with reduced mean vesicle size compared to lower intensities (Oliveira et al. 2018). This suggests that aerobic exercise intensity modulates EV size. The shift in EV size also appeared to be sex dependent. In a single-bout treadmill protocol (60% VO_2_ max for 30 min or until exhaustion), post-exercise small EV levels were lower in females compared to males, while large EV levels were higher (Rigamonti et al. 2020), suggesting that biological sex influences the size distribution of EVs released in response to aerobic exercise (Durrer et al. 2015).

Exercise-induced EV release exhibits temporal dynamics during aerobic exercise. EVs surge into circulation early, before reaching the individual anaerobic threshold, marked by a rise in lactate levels, peaking mid-exercise (15–21 min during incremental cycling), then declining towards baseline within 90 min recovery (Frühbeis et al. 2015). This pattern suggests that EVs mediate acute systemic signaling rather than sustained adaptation. Moreover, protocol design influences the magnitude of EV content and profile adaptation. Cycling triggers a sharp, transient EV spike, cleared rapidly post-exercise, while treadmill running induces a more moderate, sustained rise through early recovery, possibly due to greater eccentric muscle stress and heart rate demands (Frühbeis et al. 2015). Higher aerobic intensities further complicate this adaptation, with peripheral blood lymphocytes apoptosis leading to increased circulating apoptotic bodies, inflating circulating EV counts (Mooren et al. 2002, Battistelli et al. 2020).

EV release during resistance exercise appears more variable and protocol-dependent, often showing minimal changes in total circulating EV concentration (Kargl et al. 2024). For example, acute resistance protocols, such as 6 sets of 10 squats at 75% 1-RM (Conkright et al. 2022) and 5 days of simulated military operational exercises (Conkright et al. 2022), elicited no significant increases in EV concentration, likely due to resistance exercise’s localized metabolic stress vs. aerobics’ systemic demands. In men performing a single-bout of flywheel resistance exercise (5 sets of 10 repetitions with maximum strength), circulating EV levels increased approximately 2 fold (no size change) (Annibalini et al. 2019), while a single-bout squat protocol (6 × 10 reps at 75% 1-RM) reduced EV size in men (shifting toward small EVs) but not in women (Conkright et al. 2022). Interestingly, women exhibited higher concentrations of muscle-derived EV (SGCA⁺) than men, potentially estrogen-driven (Conkright et al. 2022).

Sex emerges as a key modulator of EV dynamics across modalities and training intensity and duration. Women showed higher muscle-derived EV proportion after 5 days of resistance training (simulated military operational exercises), mirroring aerobic patterns and aligning with menstrual cycle peaks in platelet microparticles (Conkright et al. 2022), with higher concentrations in the luteal phase than in the follicular phase (Toth et al. 2007). This pattern suggests that EV release in women may be modulated by ovarian hormone fluctuations across the menstrual cycle (Toth et al. 2007, Jonge 2003), although further studies are needed to establish the magnitude and biological significance of these effects. Longer-term resistance training (3 times/week, 60–90 min per session for 12 weeks) produced no mean change in EV size between sexes, but significantly changed EV subpopulations and total concentration, with increases observed only in men, reinforcing sex-specific adaptation to exercise (Kargl et al. 2024). Unlike acute resistance bouts, chronic training did not increase muscle-derived EVs (SGCA + or miR-206), suggesting these subpopulations are more sensitive to acute stimuli (Annibalini et al. 2019, Kargl et al. 2024).Women displayed a higher proportion of oxidative type I fibers than men, likely driven the sex divergence in total EVs concentration (Kargl et al. 2023).

Despite these shifts in the proportion of muscle-derived EVs, skeletal muscle remains a minor contributor to the circulating pool, which are dominated by platelet- and immune cell-derived vesicles. Exercise prescription variables (e.g., intensity, duration, volume, and training load) and biological factors such as sex appear to shape the molecular cargo of muscle-derived EVs more than their circulating abundance. These observations reinforce the concept that EV-mediated inter-organ communication is a key mechanism coordinating systemic exercise adaptations. The predominance of immune cell-derived EVs in circulation acquires particular relevance in the cancer context. For instance, aerobic exercise promotes NK cell mobilization and infiltration into tumors, a well-documented antitumor mechanism (Rundqvist et al. 2020, Pedersen et al. 2016), raising the possibility that NK cell-derived EVs represent an underexplored but mechanistically compelling mediator of exercise-induced anticancer effects. This would position immune cell-derived EVs as active molecular effectors of the antitumor response, a hypothesis that warrants experimental investigation.

### The effect of exercise on EVs’ molecular cargo composition

Exercise alters EV molecular cargo in a modality-, intensity-, and time‑dependent manner, turning them into dynamic carriers of exerkines that drive systemic adaptations upon target cell uptake.

A single bout of endurance exercise (treadmill running for 45 min at 50% of VO_2_max) enriched plasma EVs with SOD3 and ATP7A, thereby enhancing H_2_O_2_-dependent angiogenesis through promotion of endothelial cells migration and capillary formation in recipient vascular cells (Abdelsaid et al. 2022). Progressive cycling (60 min: 30 min 55%, 20 min 70%, 10 min 80% VO₂max) loaded EVs with 35 myokines/glycolytic proteins of muscle origin (Whitham et al. 2018), a cargo with the potential to modulate metabolic reprogramming and energy sensing in recipient cells. High-intensity interval exercise (> 90% VO₂max) modulated more than 300 EV-associated proteins (Vanderboom et al. 2021), mostly leukocyte-derived, including MMP-9 capable of degrading basement membrane components, reshaping the vascular/tumor microenvironment. In obese and type 2 diabetic individuals, a single-bout of ergometer cycling (60 min at 70% VO_2_max) increased CD36+/FATP4 + muscle-derived EVs, carrying two membrane transporters that facilitate long-chain fatty acid uptake across the plasma membrane, thereby enhancing fatty acid oxidation in recipient metabolic tissues even under conditions of chronic metabolic stress (Nielsen et al. 2019, Jeppesen et al. 2011).

**Fig. 1:**
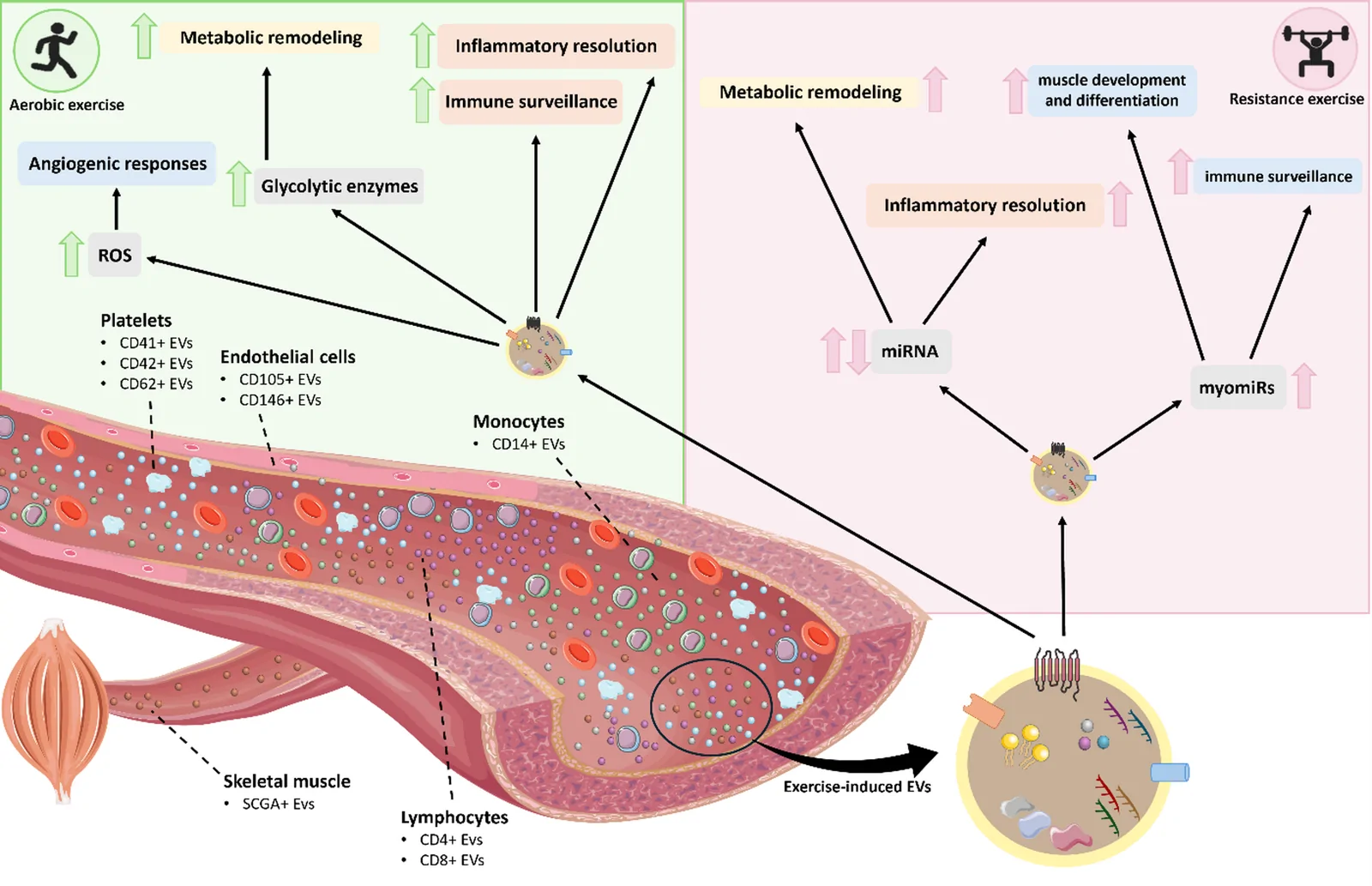
Overview of exercise-induced extracellular vesicles (EVs) released into the circulation from multiple cellular origins and the EV surface markers from platelets (CD41⁺, CD42⁺, CD62⁺), endothelial cells (CD105⁺, CD146⁺), lymphocytes (CD4⁺, CD8⁺), monocytes (CD14⁺), and skeletal muscle (SGCA⁺), and overview of the impact of exercise on EV molecular cargo, secreted in response to different types of exercise training programs. Resistance exercise is represented in red, aerobic exercise in green. This figure was partly generated using Servier Medical Art, provided by Servier, licensed under a CC BY 4.0 (https://creativecommons.org/licenses/by/4.0/)

Although research has mainly focused on aerobic exercise, resistance exercise generates distinct EV molecular signatures. High-intensity one-legged knee extension (3 sets of 10 reps at 70% RM, with 1 min rest between sets) modulated seven proteins in EVs, with leukocytes showing a greater contribution than skeletal muscle, and MMP-9 enrichment supporting repair/vascular remodelling (Vanderboom et al. 2021). Furthermore, back squats (6 sets of 10 reps at 75% RM) altered 34 miRs contained in EVs targeting growth/metabolism (IGF-I, mTOR, and PI3K/AKT) and inflammation (TGF-β, IL-6, IL-10) pathways in recipient cells (Conkright et al. 2024). These miRs may be involved in downstream processes related to metabolic remodeling and inflammatory resolution. Flywheel squats (5 sets of 10 reps at maximum strength) enriched EVs with two muscle specific miRs (myomiRs: miR-206 involved in muscle differentiation and miR-146a in immune responses), suggesting a role for these EVs in muscle development and immune surveillance (Annibalini et al. 2019, Siracusa et al. 2018). In postmenopausal women, a graded cycling protocol (initiated at 20 watts, increasing by 20 w*atts*/min until exhaustion), estrogen therapy users exhibited distinct and temporally dynamic EV miR profile: miR-191, miR-146b and miR-223 were upregulated immediately after exercise (targets implicated in the regulation of inflammatory signaling and immune cell differentiation), while one hour after exercise only miR-486 remained upregulated, a miR associated with PI3K/Akt pathway modulation, and miR-340 became downregulated in EVs, a regulator of oxidative stress and cellular senescence (Karvinen et al. 2023). Figure 1 summarizes the main alterations in EV molecular cargo induced by aerobic and resistance exercise, highlighting their tissue of origin and biological effects.

Compared to protein cargo, which tends to exert rapid, direct enzymatic or structural effects on recipient cells, EV-encapsulated miRs act post-transcriptionally, simultaneously silencing multiple target mRNAs and reprogramming gene expression networks in recipient cells over a broader temporal window. This distinction is functionally significant: while exercise-induced EV proteins may acutely modulate receptor signaling, metabolic flux, or extracellular matrix remodeling in target tissues, miR cargo has the potential to produce sustained transcriptional reprogramming, including modulation of inflammatory, proliferative, and survival pathways, whose biological consequences may outlast the exercise stimulus itself (Filipowicz et al. 2008).Overall, aerobic and resistance exercise produce distinct EV proteomes and miR profiles that mirror their physiological demands. Aerobic exercise enriches circulating EVs with metabolic enzymes (glycolytic and oxidative phosphorylation machinery), antioxidant proteins (SOD3) and angiogenic factors (VEGF, MMP-9), collectively driving systemic metabolic reprogramming, oxidative adaptation, and vascular remodeling (Abdelsaid et al. 2022, Nielsen et al. 2019, Safdar et al. 2016). Resistance exercise, by contrast, prioritizes EVs carrying myogenic miRs (miR-1, miR-133 and miR-206) and regulatory proteins targeting mTOR, PI3K/AKT, TGF-β and IL6 signaling axes, supporting muscle hypertrophy, tissue repair and immune surveillance in recipient cells (Vanderboom et al. 2021, Conkright et al. 2024, Gonzalez 2016). Biological sex and hormonal status introduce a further layer of specificity in EV quantity and composition (Rigamonti et al. 2020, Kargl et al. 2024, Noren Hooten et al. 2022), representing an additional source of inter-individual variability. These modality- and sex-specific cargo divergence position EVs as context-sensitivity exerkine vectors, with potential relevance to modulate the tumor microenvironment and inform precision exercise oncology.

## The effects of exercise-mobilized EVs on cancer progression

Exercise-mobilized EVs offer a promising counter-regulatory mechanism against cancer’s metabolic reprogramming, a hallmark sustained by tumor-derived EVs. Cancer-associated fibroblasts (CAFs) and tumor-associated macrophages (TAMs) release EVs carrying myeloid-specific lncRNA (e.g. HISLA) that stabilize HIF-1α in cancer cells, promoting glycolysis, apoptotic resistance and chemoresistance (Chen et al. 2019). In contrast, aerobic exercise-derived EVs are enriched with oxidative phosphorylation (OXPHOS) and glycolytic regulators, including pyruvate kinase, hexokinase and mitochondrial proteins, and miRs like miR-133b, which suppresses glycolysis (Vulczak et al. 2020, Aveseh et al. 2015). Therefore, EVs mobilized by exercise may regulate tumor metabolism by modulating pyruvate availability, OXPHOS activity, and reactive oxygen species (ROS) levels, and sensitizing tumors to oxidative stress-induced therapies like doxorubicin (D’Souza et al. 2018, Sugiyama et al. 2016). Overall, exercise-mobilized EVs may competitively reprogram the tumor microenvironment, antagonizing pro-glycolytic tumor-EV signaling, thereby limiting the proliferative and invasive potential of tumor cells.

**Fig. 2:**
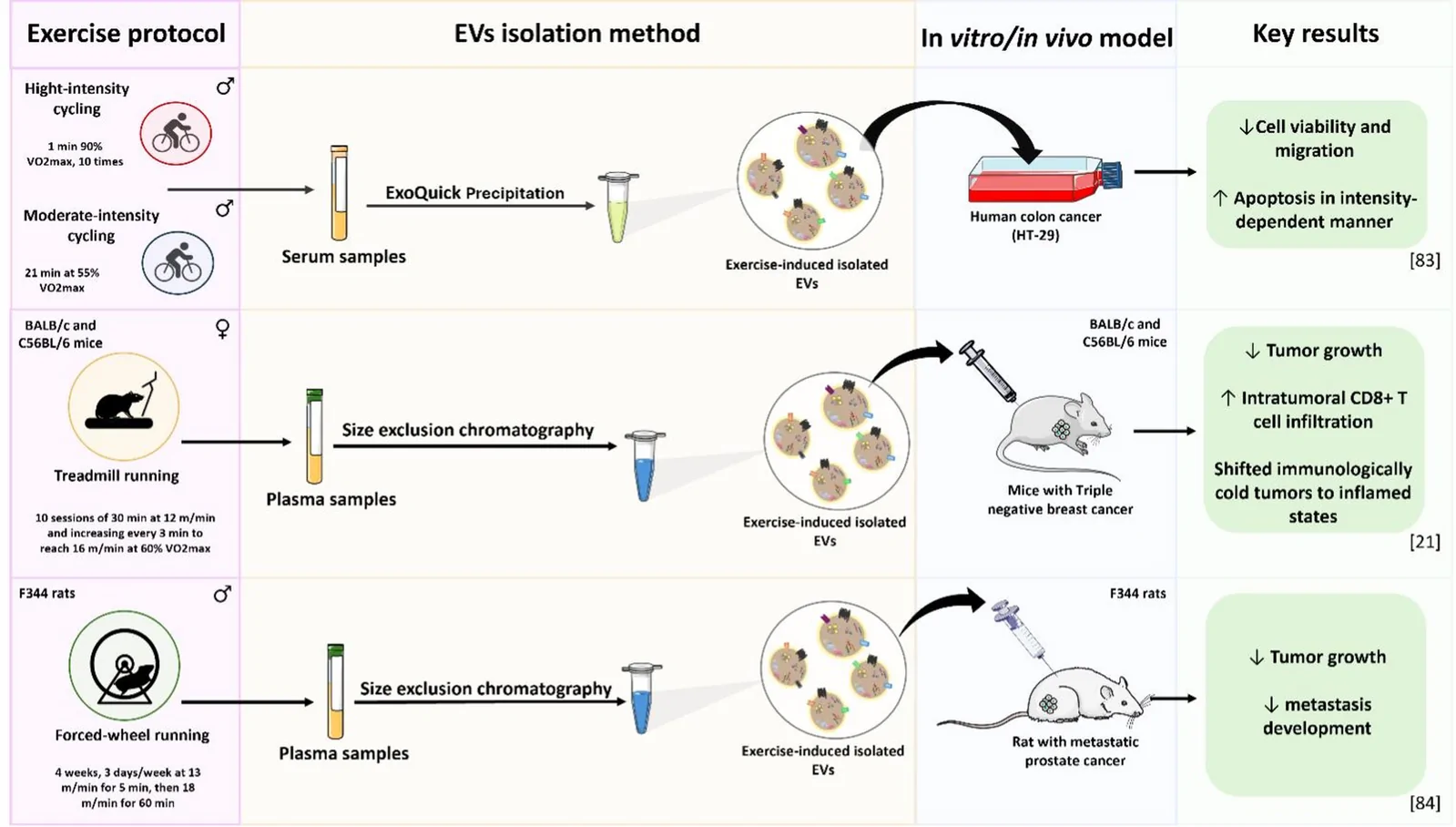
Studies evaluating the effects of different exercise protocols on circulating extracellular vesicles (EVs) and their impact on cancer progression. Three preclinical studies are summarized across four domains: exercise protocol, EV isolation method, experimental model (in vivo or in vitro), and key results on tumor microenvironment and growth. Figure was made based on experimental findings from Ozerklig et al., (Ozerklig et al. 2025), Mlynska et al., (Mlynska et al. 2025) and Sadovska et al., (Sadovska et al. 2022). This figure was partly generated using Servier Medical Art, provided by Servier, licensed under a CC BY 4.0 (https://creativecommons.org/licenses/by/4.0/)

EVs released in response to exercise have showed potential anticancer effects in preclinical models, which may support their putative role as mediators of exercise benefits. In a randomized crossover trial, plasma EVs from healthy young men post high-intensity (10 time, 1 min intervals at 90% VO_2_max, separated by 75 s of active recovery at 60 watts) or moderate-intensity (21 min at 55% VO_2_max) cycling reduced human colon cancer (HT-29) cell viability and migration while inducing apoptosis in an intensity-dependent manner (Ozerklig et al. 2025). The anticancer potential of EVs released post-exercise has also been evaluated in vivo. Circulating EVs collected from BALB/c and C56BL/6 mice after treadmill running (10 sessions of running 10 m/min warm-up, 30 min at 12 m/min and increasing by 0.5 m/min every 3 min to reach 16 m/min at 60% VO_2_max) were administered intravenously in tumor-bearing mice, in doses of 5 × 10^9^ particles in 100 µL of PBS (2.5 × 10^11^ particles/Kg; as preventive treatment a total of 5 doses, one every two days starting 9 days before tumor implementation and as therapeutic treatment a total of 5 doses, one every two days starting 7 days after tumor implementation consisting of EO771 in C57BL/6 and 4T1 in BALB/c mice) and increased intratumoral CD8^+^ T cell infiltration, delayed tumor growth and shifted immunologically cold tumors to inflamed states (Mlynska et al. 2025). These findings suggest that exercise-mobilized EVs may potentially mediate the remodeling of tumor immune microenvironment by favoring CD8^+^ T cell infiltration, thereby hypothesizing a link between the antitumor effects of exercise and exercise-mobilized EVs, although the molecular cargo responsible for these effects remains unknown (Gomes-Santos et al. 2021, Mlynska et al. 2025). In another study, EVs collected from exercised rats (forced running wheel for 4 weeks, 3 days/week at 13 m/min for 5 min, then 18 m/min for 60 min) were administered intravenously in a syngeneic orthotopic rat model of metastatic prostate cancer (100 µL of PBS containing 1.5 × 10^10^ particles, 5 days after tumor implementation, every second day for 6 weeks) (Sadovska et al. 2022). Before being injected, EVs were characterized regarding their RNA content, which displayed a significant upregulation of 10 genes and downregulation of 10 genes after exercise compared to the ones collected before exercise. Following the administration of these EVs to sedentary animals, reduced tumor growth and metastasis were observed (Sadovska et al. 2022). Although, the EV molecular content seemed to be associated with metabolic processes, protein maturation and degradation, cell survival, and immune regulation, none of the identified genes were directly associated with the inhibition of tumor growth observed after EV treatment (Sadovska et al. 2022). Despite this characterization, none of the in vivo studies were able to identify the molecular cargo of the administered EV populations that associate with the observed effects on recipient cells, precluding any mechanistic inference regarding the specific signals. It is also important to note that the EV doses administered in these preclinical studies may not reflect the concentrations or specific vesicle populations circulating following exercise, and dose comparisons across species are currently lacking. Consequently, these findings should therefore be interpreted with caution, and not as evidence of pharmacological relevance in humans. Figure 2 overviews the current in vitro and in vivo studies assessing the impact of EVs secreted post-exercise on cancer progression, demonstrating their ability to attenuate tumor growth and modulate the immune microenvironment.

Overall, research suggests that EVs released after exercise, can modulate the tumor microenvironment and metabolism, attenuating tumor progression in preclinical models. No studies to date have characterized EVs from cancer patients engaged in structured exercise programs, representing a critical gap between preclinical evidence and clinical translation.

## Conclusions

EVs released from skeletal muscle, immune cells and other tissues in response to exercise have emerged as promising mediators of the systemic anticancer effects of physical activity, with preclinical evidence implicating them in the modulation of tumor vascularization, metabolic reprogramming and immune surveillance. However, the translational relevance of these findings remains critically limited, as no clinical trials have yet characterized the composition or biological activity of EVs isolated from cancer patients undergoing exercise interventions. Such characterization faces substantial methodological and clinical challenges, including the isolation of EVs from a highly heterogeneous circulating milieu already enriched with tumor-derived vesicles, the confounding influence of comorbidities and anticancer treatments on EV biogenesis and cargo, the practical constraints of exercise interventions in patients with reduced functional capacity, and the lack of standardized protocols for EV isolation and characterization in clinical settings.

Beyond this, several conceptual and methodological challenges remain unresolved, including the relative contribution of EVs from different cellular origins to the observed anticancer effects. The extent to which aerobic versus resistance exercise protocols, different intensities and training volumes generate distinct EV populations with divergent biological activities also warrants investigation, and may raise the hypothesis that exercise modality-specific EV signatures may contribute differentially to the anticancer outcomes, as does the question of whether these responses are sex-specific or altered by tumor type, stage and treatment status.

Moreover, the EV profile following resistance exercise remains almost entirely unexplored, representing a relevant gap given the importance of resistance training in preserving muscle mass and function in cancer patients, a population particularly vulnerable to sarcopenia and/or cachexia. Furthermore, the specific molecular components of EVs, whether proteins, non-coding RNAs, lipids or metabolites, that are necessary to drive antitumor effects have yet to be identified, and the mechanisms by which these molecules interact with tumor cells and the immune microenvironment remain largely unknown, limiting the potential of using EVs from trained subjects as therapeutic tools. Addressing these questions will require carefully designed studies combining standardized and modality-specific exercise interventions with state of the art EV isolation, multi-omics cargo profiling, and functional validation in clinically relevant cancer models, with the ultimate goal of establishing post-exercise EVs as actionable modulators of the anticancer benefits of exercise and potential targets for innovative cancer care.

## Data Availability

No datasets were generated or analysed during the current study.

## Abbreviations

AKT: Protein Kinase B
ALIX1: Apoptosis-Linked Gene 2-Interacting Protein X 1
ATP7A: ATPase Copper Transporting Alpha
CAFs: Cancer-Associated Fibroblasts
CD: Cluster of Differentiation
FATP4: Fatty Acid Transport Protein 4
HT-29: Human Colon Cancer Cell Line
ICAM-1: Intercellular Adhesion Molecule 1
IGF-I: Insulin-Like Growth Factor I
IL-6: Interleukin-6
IL-10: Interleukin-10
ISEV: International Society for Extracellular Vesicles
ITGB5: Integrin Beta 5
MISEV: Minimal Information for Studies of Extracellular Vesicles
MMP-9: Matrix Metalloproteinase-9
mTOR: Mechanistic Target of Rapamycin
OXPHOS: Oxidative Phosphorylation
PBMC: Peripheral Blood Mononuclear Cells
PI3K: Phosphoinositide 3-Kinase
RM: Repetition Maximum
ROS: Reactive Oxygen Species
SGCA: Alpha-Sarcoglycan
SOD3: Superoxide Dismutase 3
TGF-β: Transforming Growth Factor Beta
TSG101: Tumor Susceptibility Gene 101
VCAM-1: Vascular Cell Adhesion Molecule 1
VEGF: Vascular Endothelial Growth Factor
EVs: Extracellular Vesicles
